# Age dependent increase in the levels of osteopontin inhibits skeletal muscle regeneration

**DOI:** 10.18632/aging.100477

**Published:** 2012-08-15

**Authors:** Preeti Paliwal, Novalia Pishesha, Denny Wijaya, Irina M Conboy

**Affiliations:** Department of Bioengineering, University of California, Berkeley, CA 94720, USA

**Keywords:** inflammation, osteopontin, myogenic stem cells, muscle regeneration, macrophage

## Abstract

Skeletal muscle regeneration following injury is accompanied by rapid infiltration of macrophages, which play a positive role in muscle repair. Increased chronic inflammation inhibits the regeneration of dystrophic muscle, but the properties of inflammatory cells are not well understood in the context of normal muscle aging. This work uncovers pronounced age-specific changes in the expression of osteopontin (OPN) in CD11b+ macrophages present in the injured old muscle as well as in the blood serum of old injured mice and in the basement membrane surrounding old injured muscle fibers. Furthermore, young CD11b+ macrophages enhance regenerative capacity of old muscle stem cells even when old myofibers and old sera are present; and neutralization of OPN similarly rejuvenates the myogenic responses of old satellite cells in vitro and notably, in vivo. This study highlights potential mechanisms by which age related inflammatory responses become counter-productive for muscle regeneration and suggests new strategies for enhancing muscle repair in the old.

## INTRODUCTION

Skeletal muscle displays a robust regenerative response upon injury where muscle stem cells (satellite cells) exit quiescence, undergo activation, proliferation and fuse to form newly regenerating myofibers [1, 2]. This process is accompanied by inflammation, e.g. infiltration of immune cells, primarily macrophages, at the site of muscle injury [3]. While it has been shown that inflammation generally increases with aging, which has been attributed to impaired immune response [4-6], several previous studies have demonstrated that in the young animals inflammatory macrophages play a positive role in clearing the wound and promoting muscle regeneration [7-13].

Muscle regenerative potential declines with aging, which is largely due to the changes in the microenvironment of muscle stem cells, but a role of an altered immune response in the age-specific decline of muscle repair is not well understood in cellular or molecular terms [14-16]. With advancing age and in certain pathologies, such as muscular dystrophy, chronic and increased inflammation impairs tissue regeneration [5, 17] and in normal aged human skeletal muscle, exercise induced injury results in a different cytokine profile as compared to young tissue [4]. However, while the published data suggests that altered inflammatory response by untimely or sustained higher production of cytokines might contribute to the decline in the regenerative properties of muscle stem cells in the old, this has not been directly shown or explained at molecular level.

OPN, a pleiotropic cytokine (also known as Spp1), is expressed in variety of tissues including macrophages and bodily fluids and broadly regulates cell migration, adhesion, immune responses and inflammation [18-21]. It has also been shown to play a key role in a number of pathophysiological conditions such as, Duchene muscular dystrophy, autoimmune diseases, cancer, tissue injury, fibrosis and delayed wound healing [18, 20, 22-25]. It was suggested that transient up-regulation of OPN after muscle injury plays a positive role in overall regeneration [26]; however, in cultured myoblasts OPN has been shown to inhibit cell migration and differentiation under some experimental conditions [27] and in pathologically inflamed muscle of MDX mice (animal model of DMD), OPN was shown to inhibit muscle regeneration [24]. Also, mice lacking OPN are healthy and normal, including skeletal muscle, and are protected against inflammatory disorders, which suggest that the absence of OPN does not influence myogenesis negatively [28].

Considering these interesting and somewhat contradictory data on the role of OPN in myogenesis compounded by the lack of information on the age-specific effects of inflammatory cytokines on muscle repair, we investigated a potential involvement of OPN in the acquired decline of muscle regeneration in the old. We found that as compared to young, OPN become over-pronounced in macrophages that infiltrate old injured muscle (at both protein and mRNA levels). Very interestingly, OPN is also elevated in the blood serum of old mice, and only if animals are injured, suggesting that this cytokine can potentially deregulate regenerative responses not only at the site of the injury, but systemically. Furthermore, we found that physiological OPN directly inhibits the regenerative responses of old muscle stem cells and ectopic OPN is able to inhibit myogenic capacity of young muscle stem cells. Notably, regeneration of old injured muscle was significantly enhanced by the neutralization of OPN and young intramuscular CD11b+ macrophages also enhanced the myogenic responses of old satellite cells in the presence of old myofibers and old serum, suggesting that secretome of young inflammatory cells is capable of negating the inhibitory influence of old stem cell niches. Summarily, this work improves our understanding of the role of inflammation in tissue aging and introduces new avenues for restoration of muscle repair in the old.

## RESULTS

### Osteopontin expression is increased in old macrophages upon muscle injury

**Figure 1:**
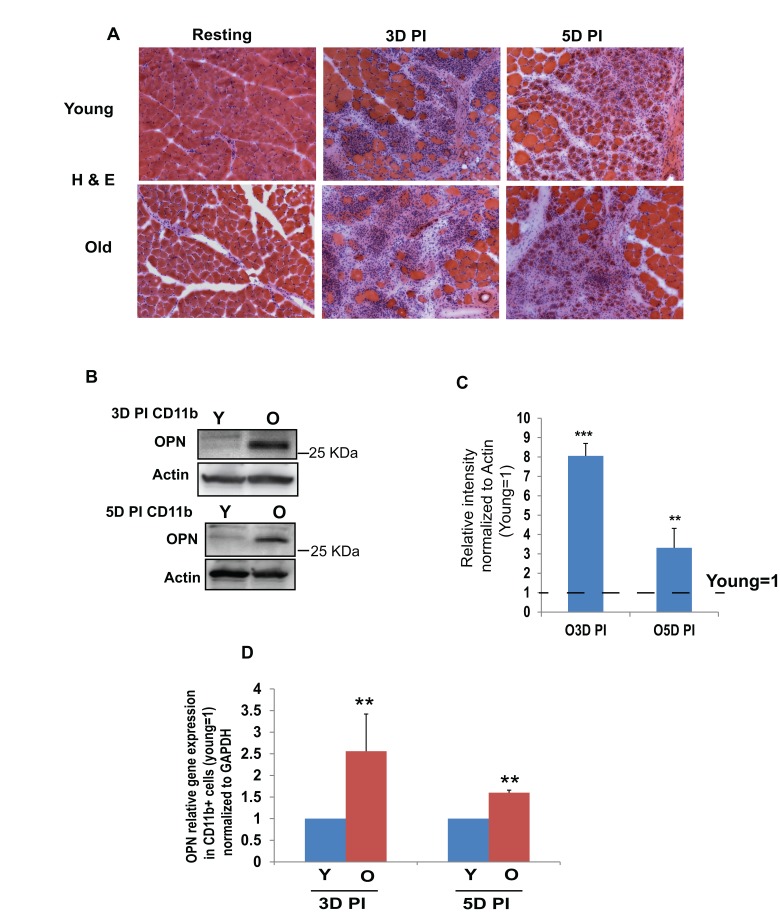
Old intramuscular CD11b+ cells produce elevated levels of OPN as compared to young (**A**) TA muscle of young (2-4 months) and old (22-24 months) C57BL6/J mice (resting and injured by cardiotoxin, CTX) was isolated at indicated days of post injury (DPI), was sectioned (10 μm) and immuno-stained with Hematoxylin and Eosin (H&E). (**B**) OPN protein (~30 KDa) was detected by western blotting in young and old CD11b+ cells that were isolated from injured young and old muscle at 3 days and 5 days post injury (DPI); (**C)** OPN was found to be significantly higher in the old cells, as compared to young (OPN is normalized to actin; young =1). (**D**) OPN-specific qRT-PCR was performed on CD11b+ cells that were isolated from young and old injured muscle at 3 and 5 DPI. Sustained increased expression of OPN mRNA was observed at both time points in old CD11b+ cells (n=3-5, p**≤0.05).

To analyze age specific changes in muscle regeneration, Young and old Tibialis Anterior (TA) and Gastrocnemius (Gastroc) muscles were injured by cardiotoxin (CTX) and compared by tissue cryosections at different time points after injury. As seen by H&E staining, pronounced myofiber disintegration (Figure 1 A) and infiltration of mononucleated CD11b+ inflammatory cells (Figure S1 A) into the injury site were observed at 3 days after injury irrespective of animal age. CD11b has been extensively used as macrophage marker [7, 15]. In complete agreement with previously published work [30] while young muscle was robustly repaired at 5 days post injury (5DPI) with new regenerating myofibers, tissue repair was significantly worse in the old mice, with fewer newly formed myofibers and evident fibrosis (Figure 1 A).

Macrophage population is predominant in the injured muscle [31] and OPN is known to be expressed in a variety of cell types including macrophages [32-34]. Hence, we hypothesize that OPN, which becomes elevated in a muscle of MDX mice [24] and in the injured muscle of wild type mice [26], might play an important role in physiological muscle aging and compared the levels of this cytokine in the intramuscular macrophages of young versus old mice. We isolated CD11b+ macrophages from young and old muscle (injured for 3 and 5 days), using magnetic bead column (MACS). This method yielded ~90% cell purity (Figure S2 A). Using Western blotting, we found approximately 8-fold increase in ~30 KDa OPN in CD11b+ macrophages that have been isolated from old, as compared to young muscle (Figure 1 B and C). OPN undergoes extensive posttranslational modifications and enzymatic cleavage at different sites, which gives rise to various cleaved forms ranging from ~25-75 KDa; such OPN variants regulate diverse cell behavior and expression of multiple genes [35, 36]. To confirm the age-specific increase in OPN, we also performed qRT PCR studies and found that OPN mRNA was significantly elevated in CD11b+ cells that were isolated from old injured muscle, as compared to young (Figure 1 D). FACS analysis quantitatively determined that the numbers of CD11b+ inflammatory leukocytes are similar in the injured muscles of young and old mice (Figure S1 B).

These data suggest that while the numbers of CD11b+ cells that infiltrate injured skeletal muscle do not significantly change with age, OPN expression becomes elevated in the intramuscular inflammatory macrophages of old mice, as compared to young.

### OPN is increased in the serum and myofiber niche of injured old mice

**Figure 2:**
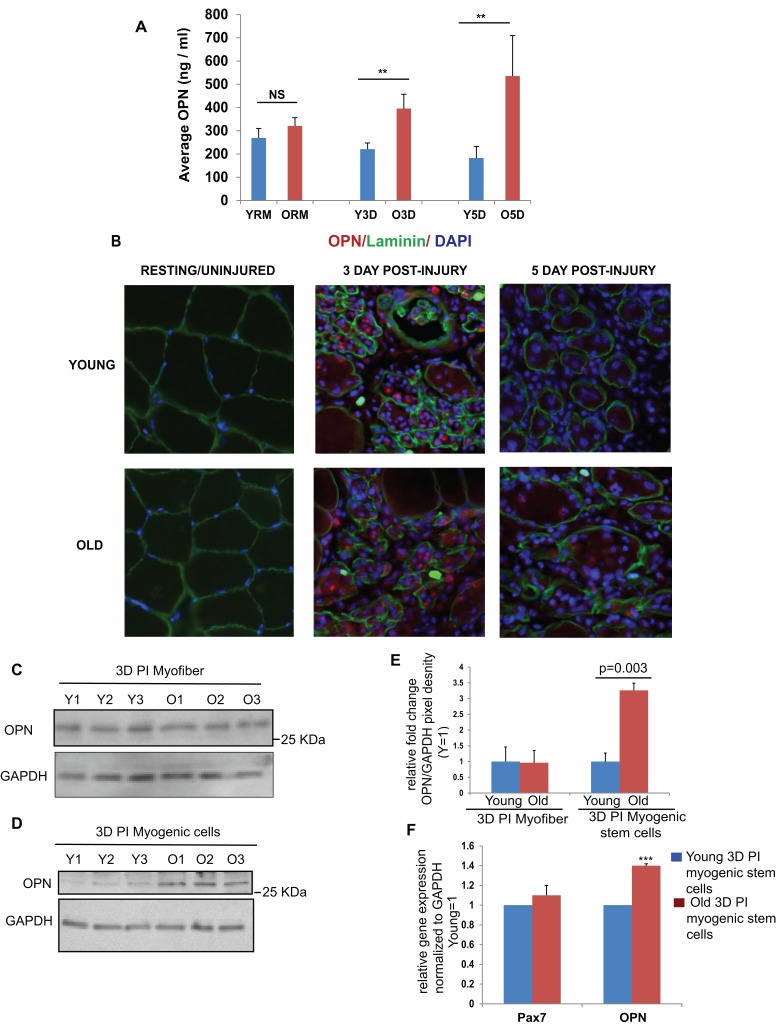
Increased levels of osteopontin in serum and myofiber niche of old mice upon muscle injury (**A**) Blood sera were collected from young and old mice, either non-injured or at 3 days and 5 days after muscle injury and OPN levels were quantified by ELISA. Resting young and old blood serum levels do not show significant age-specific difference, while both at 3 days and 5 days after muscle injury old mice have higher levels of serum osteopontin than young (n=3-6, ± SEM, p**≤0.05). (**B**) Tibialis Anterior (TA) muscle sections from uninjured (resting), 3DPI and 5DPI muscle were co-immunostained for Laminin (to visualize the myofiber periphery) and OPN. OPN is undetectable in the uninjured skeletal muscle or young and old mice. In contrast, at 3 days post injury both young and old muscles show pronounced OPN at the site of injury and old muscle has considerably more OPN than young. By 5 days post injury, OPN becomes greatly diminished at the site of injury / regeneration in young muscle, but old muscle with its poor repair displays sustained OPN presence. At 3 days post injury myofibers (**C**) and myogenic stem cells (**D**) obtained from young and old mice were analyzed for OPN expression by western blotting and qRT-PCR (**F**) Old myogenic stem cells expressed higher levels of OPN as compared to young both at protein and mRNA levels, while the levels of OPN did not change with age in myofibers. (quantified in **E;** n=3, ± SEM, p**≤0.05).

OPN is a secreted molecule that is present in soluble form in many bodily fluids including milk, blood and urine [21, 22]. To analyze the age specific systemic changes in OPN, ELISA was performed on serum obtained from young and old mice (resting or after muscle injury). In correlation with the high levels of OPN produced by the old intramuscular CD11b+ cells, serum levels of OPN were also found to be increased with age and interestingly, only when old mice were injured (Figure 2 A). These results suggest either that CD11b+ cells, which infiltrate injured muscle of old mice secrete elevated levels of OPN into blood circulation or that other cells in the old injured animals contribute high levels of soluble OPN into blood sera.

To confirm and extrapolate these results and to determine whether the production of OPN by CD11b+ cells and / or elevated levels in blood serum translate into higher levels of this cytokine in the local niches of old muscle stem cells, we performed co-immunostaining of OPN with laminin (a key component of myofiber basement membrane) in 10 μm muscle cryosections. As shown in Figure 2 B, we found more OPN in the ECM of old injured muscle, as compared to young (particularly, at 5 DPI). Resting muscle that does not have infiltrating inflammatory leukocytes did not have detectable OPN in either young or old animals (Figure 2 B).

To analyze whether old muscle cells (themselves) up-regulate production of OPN, we performed western blotting on the lysates of injured myofibers and muscle stem cells. As shown in Figure 2 C (quantified in Figure 2 E), no statistically significant age-specific elevation of OPN was detected in muscle fibers; however, as compared to young, higher levels of OPN were observed in the old satellite cells (Figure 2 D, quantified in Figure 2E). As previously published, our satellite / myogenic cell isolation method yields high cell purity [37] which we also confirmed here for both young and old cells by Pax7 immunostaining (Figure S2 B). Further, ~1.5 fold increase in OPN mRNA levels was seen in old myogenic stem cells when compared to young (Figure 2 F).

These data demonstrate that OPN becomes persistently elevated in the local and systemic niches of old muscle stem cells after tissue injury.

### Age-specific elevation in OPN inhibits myogenic responses

**Figure 3:**
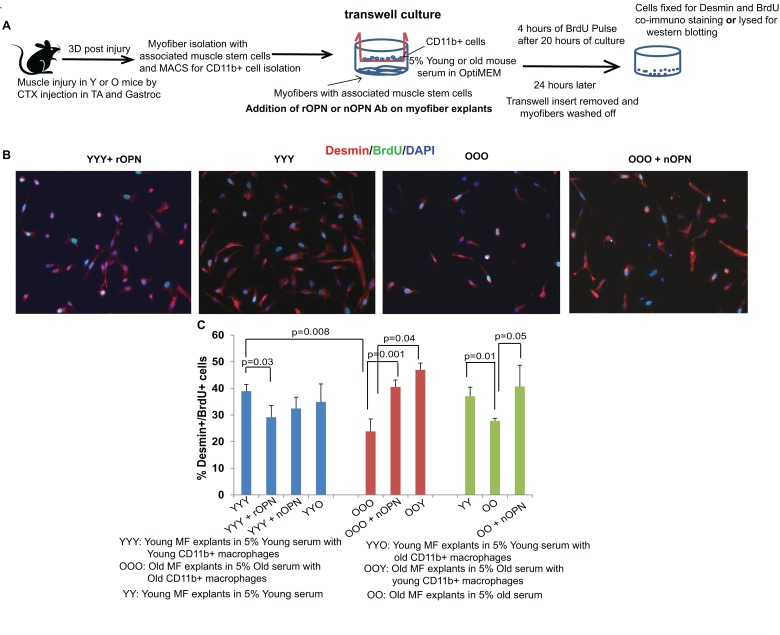
Osteopontin inhibits regenerative responses of muscle stem cells in co-cultures with CD11b+ cells (**A**) Schematic representation of transwell culture assay. Myofiber explants (satellite cells associated with myofibers) were obtained from 3 DPI young and old TA, were plated on ECM coated lower chamber of transwell in their respective 5% serum whereas MACS isolated CD11b+ macrophages from the same injured muscles of young and old mice were isochronically or hetrochronically plated on 0.3 μm PE membrane transwell insert. At the time of plating, these co-cultures were treated (or not) with rOPN or nOPN antibody, and after 20 hours, co-cultures were pulsed with BrdU for 4 hours and subsequently, fixed and co-immunostained for BrdU and Desmin. (**B**) Representative pictures of YYY (Young myofiber in Young mouse serum with Young CD11b+ macrophages), YYY + rOPN (recombinant OPN), OOO (Old myofiber in old mouse serum with Old CD11b+ macrophages) and OOO + nOPN (neutralization of OPN) are shown. (**C**) Quantification of the myogenicity in the above-described transwell co-cultures. Bar graphs represent the mean and standard deviation in percent of BrdU+/Desmin+ proliferating myogenic cells; n=3, ±S.E.M. Young macrophages or neutralization of OPN increased the clonogenic / myogenic capacity of old satellite cells that associate with old myofibers and cultured in old serum.

To test the functional significance of the uncovered age-specific up-regulation of OPN and to analyze the specific role of macrophages, we performed co-cultures of myofibers with their associated muscle stem cells and CD11b+ macrophages in transwell plates. This system allows the transport of secreted molecules from two different compartments but does not permit direct cell-cell contacts between macrophages and either myofibers or the myogenic cells (Figure 3 A). In this experiment, young and old satellite cells were cultured with their own local niche (myofiber) and in their own serum, but the age of the CD11b+ cells was either isochronic (young for young and old for old) or heterochronic (young for old and old for young) (Figure 3 A). Importantly, OPN was neutralized in some of the co-cultures and exogenous OPN was added to some of the co-cultures (Figure 3 A). The regenerative capacity of satellite cells was determined based on their ability to quickly (overnight) generate colonies of desmin+/BrdU+ myogenic cells progenitor cells [30].

As shown in Figure 3 B and C, young satellite cells cultured with young serum and young macrophages (YYY) produced many more myogenic colonies than old satellite cells cultured with old serum and old macrophages (OOO). Remarkably, presence of young CD11b+ cells rejuvenated myogenic responses of old satellite cells, even when old serum and old myofibers were present (OOY); and neutralization of OPN enhanced myogenicity of old satellite cells in co-cultures with old myofibers, old serum and old CD11b+ cells (OOO+ nOPN) (Figure 3 B and C). Confirming the negative role of OPN on myogenicity, recombinant OPN reduced the regenerative capacity of young satellite cells, which were cultured with young myofibers and young serum (YYY+ rOPN) (Figure 3 B and C). In agreement with the data on the elevated levels of OPN in the circulation of old injured mice, neutralization of OPN enhanced myogenesis in the cultures of old satellite cells, old myofibers and old sera (even when CD11b+ cells were not present) (Figure 3 C). Interestingly, transwell co-cultures where old macrophages were co-cultured with young myofiber explants in young serum did not show significant decrease in myogenicity of young satellite cells (YYO) (Figure 3 C). These results indicate that young myofiber niche and serum counteract the negative effect of old macrophages, which is consistent with previous report on heterochronic parabiosis [29]. Further, administration of nOPN antibody to young myofiber explants that were cultured with young serum and young macrophages (YYY+ nOPN), did not show a significant increase in myogenicity of young satellite cells (Figure 3 C), indicating that myogenic responses might be already maximal for the young muscle stem cells in context of their young local and systemic niches.

To validate these data further, we adsorbed a range of OPN concentrations into matrigel substrate of young and old myofiber explant cultures (Figure S3 A). The diminished generation of desmin+/BrdU+ colonies followed by the decrease in the numbers of eMyHC+ differentiated myotubes was observed in both young and old myofiber explants cultures that were exposed to ectopic OPN, as compared to control vehicle (Figure S3 B, C, D and E). These results substantiate the negative effects of OPN on myogenic cell proliferation and subsequent differentiation. This data also confirm that desmin/BrdU assay correctly detects proliferating fusion-competent myoblasts, which are the progeny of activated satellite cells and are committed to form eMyHC+ myotubes [30].

### Neutralization of OPN in vivo improves muscle regeneration and up regulates myogenic markers in old mice

**Figure 4:**
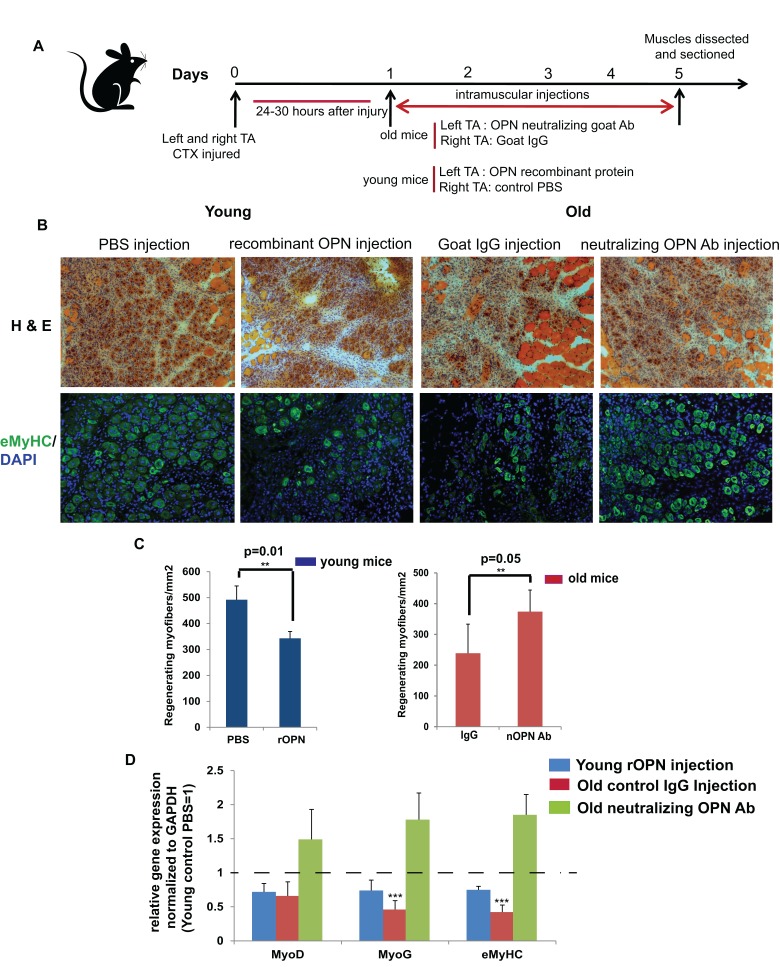
Modulation of osteopontin alters muscle regeneration in mice in vivo (**A**) Schematic representation of in vivo modulation of OPN in young and old mice upon muscle injury. TA muscles of young and old mice were injured with CTX. 24-30 hours later (and subsequently daily for total of 4 days) the injured muscles of young mice were injected with PBS (right leg) or recombinant OPN (left leg); while the injured muscle of old mice were injected in the same regiment with goat IgG (right leg) and anti-OPN antibody (nOPN) in the left leg. Muscles were dissected on day 5 and analyzed for success in regeneration in 10 μm cryosections. (**B**) Muscle sections were stained for Hematoxylin and Eosin (H&E) and immuno-stained for embryonic myosin heavy chain (eMyHC), to visualize and quantify newly regenerating myofibers; representative images are shown. (**C**) Bar graph show mean and standard deviation of the numbers of new myofibers that were quantified based on the H&E staining; n=3, p≤0.05. (**D**) RNA isolated from Tibialis Anterior (TA) muscle of young and old mice that were treated as depicted in Figure 4 A were analyzed for myogenic marker gene expression by qRT-PCR. Old control mice have decreased expression of the studied myogenic markers as compared to young control mice. In vivo neutralization of OPN in muscle of old mice significantly induced MyoD, Myogenin and eMyHC myogenic markers, making their expression similar to that of young mice, and accordingly, the over expression of OPN in young mice considerably decreased the levels of these myogenic markers. Levels of young control mice (treated with PBS) were set as 1 (n=3 ±SEM p**≤0.05).

The anti-myogenic effects of OPN that manifested in cultured satellite cells prompted us to test if neutralization of OPN would enhance and exogenous OPN would inhibit muscle regeneration in vivo. TA muscle of young and old mice were injured with CTX and 24-30 hours later neutralizing OPN antibody was injected intramuscularly into left TA of old mice, recombinant OPN was injected into left TA of young mice, while control Goat IgG and PBS were injected into right TAs of these animals daily for 4 days (Figure 4 A). TA muscles were dissected from young and old mice on day 5 post injury and assayed for the numbers of newly formed myofibers by H&E staining and by the immunofluorescence for eMyHC in 10 μm cryosections (Figure 4 B and D). As compared to control injections, neutralization of OPN in old mice enhanced muscle regeneration by 25%, while exogenous OPN decreased the regenerative capacity of young muscle by 20%, (Figure 4 C and D). Consistently with these results, neutralization of OPN significantly increased the levels of MyoD, Myogenin and eMyHC in the injured / regenerating muscle of old mice whereas exogenous OPN moderately decreased MyoD, Myogenin and eMyHC gene expression in the muscle of young mice (Figure 4 D). These results suggest that attenuation of OPN can enhance regeneration of aged muscle, while exogenous OPN inhibits repair of young muscle in vivo.

## DISCUSSION

Skeletal muscle injury is accompanied by rapid infiltration of immune cells, where macrophages play a prominent role in successful tissue regeneration [8, 12]. With age, the muscle environment dramatically changes and becomes unsupportive for the regenerative responses of satellite cells [5, 38, 39]. Multiple age-specific differences in the niche of satellite cells have been implicated in the decline of old muscle repair, however, relatively little is known about the role of altered inflammatory response in the aging of tissue regeneration either in general or in skeletal muscle.

This work is the first to analyze the age-specific changes in OPN and the effects of this cytokine on organ stem cells. Our studies show that levels of OPN in the inflammatory CD11b+ leukocytes, in blood serum and in the muscle ECM all become altered with age in ways that impair muscle repair. Notably, all these age-specific changes in the levels of OPN are detected only when tissue is injured, hence they are directly relevant to the connection between the immune response (cytokine response) to an injury and the age-specific effectiveness of tissue repair. Of particular relevance is the elevation of OPN in the circulation of injured old mice, because it suggests that in an old animal the inhibitory effects of OPN on the regeneration of skeletal muscle (and perhaps, other organs) would propagate systemically once local tissue injury has occurred.

The role of OPN in inflammation has been well documented, but it also regulates a variety of cell behaviors due to its pleiotropic nature [18-21, 40]. In skeletal muscle, OPN has been shown to increase early after muscle injury and rapidly decline thereafter [26]. Consistent with this report, we were unable to detect OPN expression in resting muscle and detected it in the vicinity of necrotic myofibers when tissue was injured. Earlier reports have also shown that OPN is increased in T cells in dystrophic mice [24], which in the light of our data suggests that immune cells that infiltrate both: pathological and physiologically old tissues over-produce OPN. Such elevated levels of OPN would impose inhibition on the regenerative performance of satellite cells that are resident to the aged muscle and likely would also block the myogenicity of satellite cells in young pathological tissue, since based on our results OPN directly inhibits muscle stem cell proliferation and subsequent differentiation not only of old, but also of young muscle progenitor cells. In agreement with our conclusions, increased levels of OPN have been implicated in many pathophysiological disorders and as a cause of tissue fibrosis; and in the mouse model of muscular dystrophy (MDX mice) where inflammation becomes exacerbated with time, OPN knockout improves tissue health [20, 23-25, 41].

Molecular mechanism(s), by which pleiotropic cytokine OPN inhibits the regenerative responses of muscle stem cells remain to be determined, but the data on improvement of old muscle regeneration and enhanced expression of myogenic markers upon neutralization of OPN demonstrate the cause and effect connection between the elevation of OPN and the lack of muscle regeneration in the old and suggest new therapeutic strategies for rejuvenation of tissue regeneration. The rejuvenation of myogenic responses ex-vivo by the cytokines that are produced by young CD11b+ cells in conjunction with the results demonstrating that young local and systemic niches “protect” young satellite cells against the negative effects of OPN, suggest that experimental modulation of inflammatory cytokines (exemplified by OPN) would significantly contribute to the strategies for improving regeneration of old muscle and perhaps, old injured / inflamed tissues in general. Summarily, our data suggests that OPN might be one of the key contributors to the decline of old muscle repair linking the age-specific defects in the inflammatory response to tissue injury with the poor performance of muscle stem cell in the old.

## METHODS

### Animal strains

C57BL6/J (2-3 months old) and C57BL/6J (22-24 month old) male mice were obtained from pathogen free breeding colonies at The Jackson Laboratories and NIH respectively. Animals were housed at the Northwest Animal Facility, University of California, Berkeley and procedures were performed in accordance to administrative panel on the Office of Laboratory Animal Care, UC Berkeley.

### Reagents and antibodies

BrdU, ECM and DNase1 were from Sigma while recombinant mouse osteopontin was purchased from R&D. Antibodies to BrdU (ab6326; rat monoclonal), osteopontin for western blotting (ab8848; rabbit polyclonal), GAPDH (ab 9483; goat polyclonal) and Desmin (ab 15200; rabbit polyclonal) were from Abcam. Laminin (Rat monoclonal) and Actin (rabbit polyclonal) antibody was from Sigma. CD11b (rat monoclonal), goat normal IgG and osteopontin (goat polyclonal) for immunostaining and in vivo neutralization were from R&D, Pax7 and eMyHC was from DSHB; secondary HRP antibodies were purchased from Santa Cruz. For flow cytometry, Cd11b-FITC and rat IgG2b-FITC was from eBiosciences while secondary fluorophore antibodies were from Molecular Probes, Invitrogen.

### Western blotting and ELISA

For whole muscle lysate preparation, Tibialis Anterior (TA) or Gastrocnemius (Gastroc) muscle was homogenized in Miltenyi Tissue dissociator instrument in Tissue lysate buffer (Thermo Scientific) containing 1X phosphatase inhibitor (Roche) and 1X Protease inhibitor (Roche). For cells, lysis was performed in RIPA buffer (50mM Tris-Cl pH7.6, 150mM NaCl, 0.1% SDS, 1% NP-40 containing phosphatase inhibitor and protease inhibitors). The protein concentration was determined by Bradford Assay. Lysates were re-suspended in 1X Laemmli buffer (BioRad) containing beta-Mercaptoethanol, boiled for 5 minutes at 1000C, and either run on precast 4-20% Gels or 10% Criterion gels from BioRad. The proteins were then transferred to nitrocellulose membrane (GE) for 2 hours and blocked in 5% Milk in PBS containing 0.05% Tween-20. Protein expression was visualized using ECL-Prime reagent (Amersham), detected by BioRad Gel Doc/Chemi Doc imaging system and analyzed by Image J.

For blood serum collection, young and old mice blood was collected and incubated at 370C for 15 minutes with gentle agitation. The tubes were spun down for 3 minutes at 5000 rpm and supernatant collected. For osteopontin ELISA, R&D kit was used according to manufacturer's recommendation.

### Immunofluorescence and Histological analysis

Immunofluorescence histological analysis was carried as described earlier [42]. In brief, cells were pulsed with 0.01mM BrdU for 4-5 hours, fixed with 4% PFA for 15 minutes at room temperature followed by permeabilization with 0.25% Triton-X 100 for 12 minutes. This was followed by 0.2 units/ul DNase1 treatment for 25 minutes, washed with 1XPBS and blocked for one hour in blocking buffer (1%BGS + 1% BSA + 0.1% Na-Azide in 1XPBS) followed by primary antibody incubation in blocking buffer for 2 hours or overnight and secondary antibody incubation for 1 hour in blocking buffer with Alexa fluorophore conjugated species specific secondary antibody (Invitrogen). For muscle sections, harvested TA muscle was soaked in 20% sucrose in PBS for few hours, embedded in OCT (Tissue-Tek), frozen in isopentane cooled liquid nitrogen and cryosectioned at 10 μm (Shandon cryostat). Sections were aligned on super frost plus slides and subsequently stained with Hematoxylin and Eosin to visualize areas of injury and determine muscle regenerative potential. The number of regenerative myofibers/mm2 throughout the muscle was calculated by Image J software. For immunostaining, muscle sections were post fixed with 4% PFA for 10 minutes and permeabilized with 0.25% TritonX-100 for 12 minutes and blocked with CAS (Invitrogen) for 30 minutes. The primary antibody incubation in CAS block was carried either at room temperature for 2 hours or overnight at 40C followed by respective fluorophore conjugated secondary antibodies. Cells were mounted with mounting media containing DAPI (Prolong Gold Antifade, Invitrogen) to visualize nuclei in all immunostaining experiments.

### CD11b cells isolation and Transwell culture

For CD11b+ cell isolation, 10 and 30 ul of cardiotoxin (CTX, 0.25mg/ml) was injected in TA and Gastroc of young and old mice and muscles were harvested after 3 days and 5 days post injury. Mononuclear cells were released from injured muscles by trituration after incubation in Collagenase type I (600 units/ml). This was passed through 100 and 40 μm strainer respectively and spun at 500g for 5 minutes at 40C. The pellet was resuspended in ice cold 2% BSA in PBS and blocked with Fc receptor blocking solution (Miltenyi Biotec) for 10 minutes followed by incubation with CD11b magnetic beads (Miltenyi Biotec) for another 15 minutes at 40C. The CD11b+ cells were sorted using Mini MACS column (Miltenyi Biotec) according to manufacturer recommendations. For transwell culture, CD11b+ immune cells isolated by MACS were plated isochronically or heterochronically on transwell insert of 0.3 μm PE membrane in OptiMEM medium whereas myofiber explants (triturated myofibers with associated satellite cells) were cultured on lower chamber coated with ECM in 5% isochronic mouse serum in OptiMEM with and without rOPN and or nOPN for 24 hours. The cultures were pulsed with BrdU (0.01mM) for 4 hours followed by removal of transwell insert and myofiber washing while adherent myofiber associated stem cells were fixed with 4% PFA and co-stained with Desmin and BrdU antibodies.

In vivo muscle experiments. For in vivo muscle experiments, TA muscle was injured with CTX in young and old mice and 30-40 hours after injury, recombinant osteopontin (4ug/ml) was injected in one leg of injured TA of young mice whereas control PBS was injected in other leg of same mice while neutralizing osteopontin antibody (5ug/ml) in one leg and control goat IgG in other leg was injected in old mice each day for a total period of 3 days followed by muscle dissection which were processed for RNA and protein lysate or frozen down for cryosectioning. For 10 μm sections, whole TA muscle was sectioned and stained for H&E to visualize the site of injury and newly formed regenerating myofibers were counted. All the experiments were done in 3 independent young and old mice.

### qRT-PCR assay

For qRT-PCR, RNA was isolated from MACS sorted CD11b+ cells using RNeasy purification kit from Qiagen according to manufacturer instructions. For whole muscle, tissues were kept in RNA later and homogenized using Miltenyi Biotec Tissue dissociator and further RNA was isolated using Qiagen kit. 500ug of RNA was reverse transcribed using SA Biosciences RT Kit. For qRT-PCR, cDNA obtained was diluted to 1:5 and reaction was set using BioRad iQ5 SybrMix in iQ5 BioRad cycler. All reactions were run in triplicate from at least three independent experiments and gene expression analyzed using delta Ct values. The list of primers used for qRT-PCR is provided as supplementary table S1 in supplementary section.

### Statistical analysis

At least 3 independent set of experiments were performed and student t-test was used to calculate significance *** p≤0.01 and ** p≤0.05. Data was plotted as mean ±S.E.M.

## SUPPLEMENTARY INFORMATION AND METHODS


