# Supplementary material for: Age dependent increase in the levels of osteopontin inhibits skeletal muscle regeneration
Source: Aging (Albany NY). 2012 Aug 15;4(8):553–66. doi: 10.18632/aging.100477 (PMC3461343; doi:10.18632/aging.100477)
Supplement: Supplementary file 1 [file aging-04-553-s001.pdf]

SUPPLEMENTARY INFORMATION

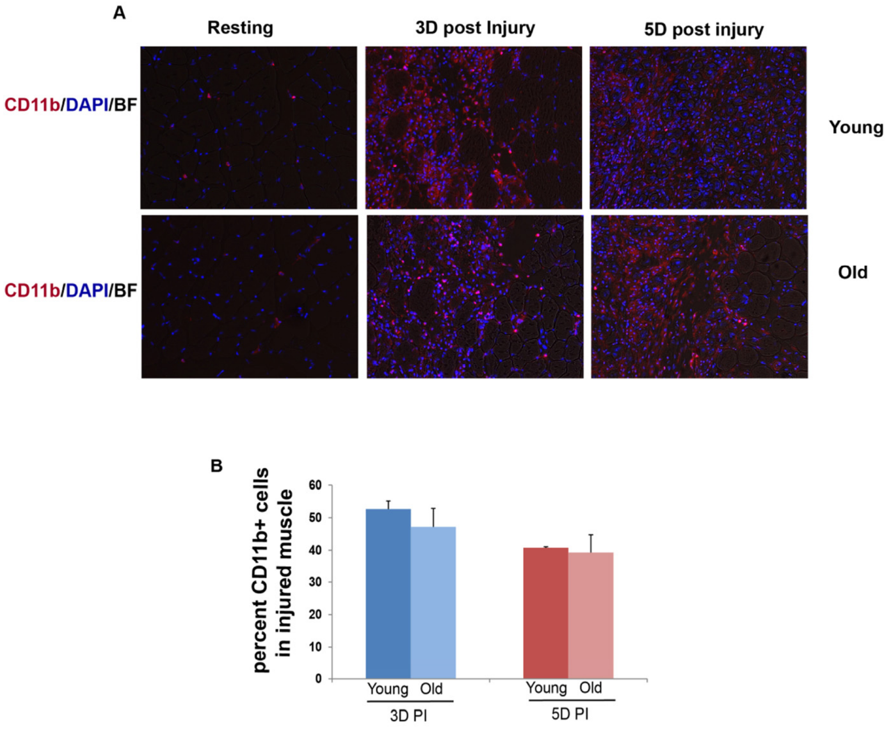

Figure S1. (**A**) **Infiltration of CD11b+ macrophages upon muscle injury.** 10 micron muscle sections from young and old mice at resting stage and different time points of CTX induced injury were stained for CD11b. Huge infiltration of CD11b+ macrophages was detected at 3 day post injury and 5 day post injury. Nuclei were stained positive with DAPI. (**B**) **CD11b+ population remains unchanged in young and old injured muscle.** A representative histogram of CD11b population in young and old 3 days and 5 days post injured muscle analyzed by flow cytometry. As compared to young, old 3 days post injured muscle showed insignificant slight decrease in CD11b population. 5 days post injured regenerating muscle show reduced CD11b population as compared to 3 days post injured muscle (data represents ±SEM, n=3 p**≤0.05).

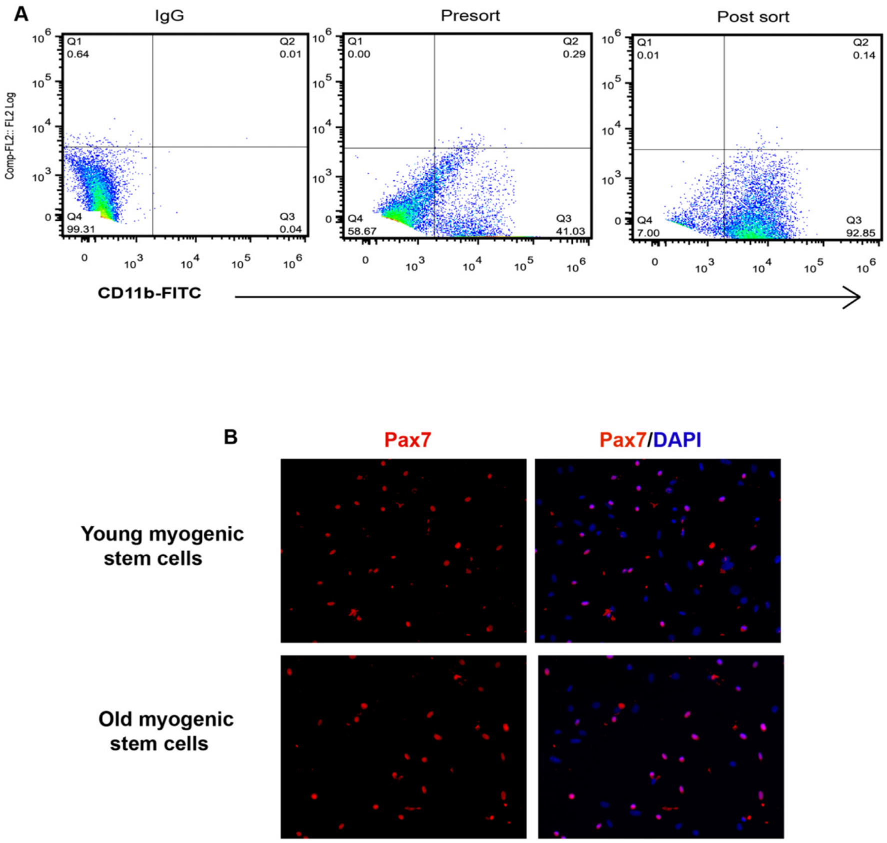

Figure S2. Flow cytometry analysis of magnetic bead sorted CD11b+ immune cells. (**A**) Young and old 3 day and 5 day CTX injured muscle were sorted for CD11b+ cells using magnetic beads and analyzed by flow cytometry to determine purity of cells. Greater than 90 % of cell purity was obtained using Miltenyi magnetic beads. (**B**) **Determination of Pax7 expression in young and old myogenic stem cell cultures.** Representative image of young and old myogenic stem cell cultures immunostained with pax7 antibody to assess the purity of stem cells obtained after isolation from injured muscle. DAPI stains nuclei.

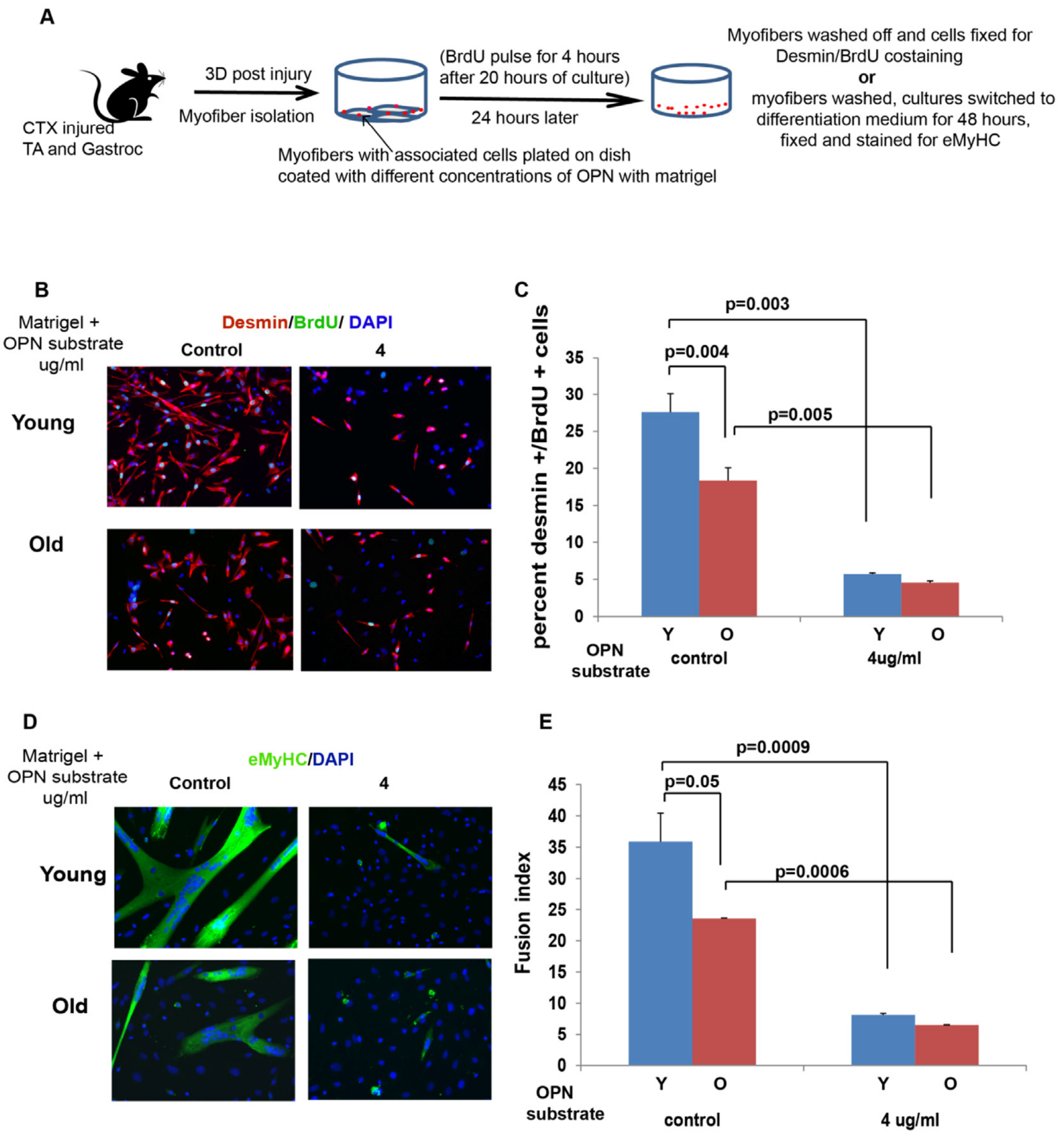

Figure S3. Exogenous osteopontin inhibits muscle stem cell proliferation and subsequent differentiation in vitro. (**A**) **Schematic representation of myofiber explant assay.** Myofiber explants (satellite cells/ muscle stem cell associated with myofibers) obtained from 3 day cardiotoxin injured young and old Tibialis Anterior skeletal muscle were plated on ECM mixed without and with recombinant osteopontin (4 ug/ml) for total of 24 hours in their respective 10% serum. The cultures were pulsed with BrdU for 5 hours and subsequently co- immunostained with BrdU and Desmin antibodies. In another condition, after 24 hours, cultures were switched to differentiation medium containing 2% horse serum in DMEM for 48 hours, fixed and stained for eMyHC to quantify newly formed myofibers. (**B**) **Representative images of Desmin +/BrdU+ proliferating cultures.** Control and 4 µg/ml OPN substrate young and old myofiber explant cultures co-stained with desmin and BrdU antibodies are shown. Addition of osteopontin inhibits proliferation of young as well old myogenic cultures. (**C**) **Quantification of percent of BrdU^{+}/Desmin^{+} cells**. Histogram represents the percent of BrdU/Desmin+ proliferating myogenic cells quantified in young and old myofiber explants with and without recombinant osteopontin. Control is no recombinant osteopontin addition. (n=3 to 5 ±S.E.M.) (**D**) **Representative images of eMyHC+ differentiated de-novo myotube cultures.** 24 hours control and 4 ug/ml OPN substrate young and old myofiber explant cultures in their respective 10% mouse serum were switched to differentiation medium for 48 hours and stained for eMyHC antibody. Addition of recombinant osteopontin as substrate subsequently inhibits differentiation of young as well old myogenic cultures. (**E**) **Quantification of percent of eMyHC^{+} myotubes.** Histogram represents the percent of eMyHC+ de-novo myotubes quantified in young and old myofiber explants with and without recombinant osteopontin a substrate. (n=3 to 5 ±S.E.M.)

SUPPLEMENTARY METHODS

Muscle injury and Myofiber explants. 10 and 30 ul of cardiotoxin (CTX, 0.25mg/ml) was injected in Tibiais Anterior (TA) and Gatsrocnemius (Gastroc) of young and old mice and muscles were harvested after 3 days and 5 days post injury. Muscles were digested in 250 units/ml of Collagenase Type II in DMEM for one and half hour at 37^0C followed by gentle trituration to release single myofibers. 3 day post injured myofiber explants from young and old mice containing activated satellite cells were plated on 8 well chambered slides coated with ECM (1:500) or ECM + recombinant mouse osteopontin (4 µg/ml) for 24 hours in their respective sera (10% in OptiMEM) followed by BrdU pulse after 20 hours. For differentiation experiment, myofiber explants after 24 hours in 10% sera were switched to differentiation medium containing 2% horse serum+ 1% Penicillin/Streptomycin in DMEM (CellGro) for 48 hours and then cells were fixed with 4% PFA for immunostaining.

Flow Cytometry. Mononucleated cells obtained after muscle trituration from young and old mice at different days of injury were resuspended in FACS staining buffer, blocked with FcY R blocking solution and stained with fluoroscent conjugated CD11b antibody. IgG-FITC served as negative control for each condition. The cells were run through BD FC500 flow cytometer and data analyzed using FlowJo software. For CD11b magnetic bead sorted cells, presort and post sort cells were stained with CD11b-FITC and subsequently analyzed to determine purity of cell population.

Supplementary Table S1. List of primers for qRT-PCR

**OPN F**	5'- TCCCTCGATGTCATCCCTGTTG -3'
**OPN R**	5'-GGCACTCTCCTGGCTCTCTTTG-3'
**Myogenin F**	5'-GACCCTACAGACGCCCACAA-3'
**Myogenin R**	5'-CCGTGATGCTGTCCACGAT-3'
**MyoD F**	5'-CGGCTCTCTCTGCTCCTTTG-3'
**MyoD R**	5'-GAGTCGAAACACGGGTCATCA-3'
**eMyHC F**	5'-AGAGGACGTGTATGCCATGA-3'
**eMyHC R**	5'-TGGCCATGTCCTCAATCTTGT-3'
**GAPDH F**	5'-GGGAAGCCCATCACCATCT-3'
**GAPDH R**	5'-GCCTCACCCCATTTGATGTT-3'
